# Esophageal Lipoma and Liposarcoma: A Systematic Review

**DOI:** 10.1007/s00268-020-05789-4

**Published:** 2020-10-07

**Authors:** Davide Ferrari, Daniele Bernardi, Stefano Siboni, Veronica Lazzari, Emanuele Asti, Luigi Bonavina

**Affiliations:** 1 Department of Biomedical Sciences for Health, Division of General and Foregut Surgery, University of Milan, IRCCS Policlinico San Donato, 20133 Milan, Italy; 2 Division of General and Foregut Surgery, IRCCS Policlinico San Donato, Piazza Edmondo Malan, San Donato Milanese, 20097 Milan, Italy

## Abstract

**Background:**

Esophageal lipomatous tumors, also reported as fibrovascular polyp, fibrolipoma, angiolipoma, and liposarcoma, account for less than 1% of all benign mesenchymal submucosal tumors of the esophagus. Clinical presentation and therapy may differ based on location, size, and morphology. A comprehensive and updated systematic review of the literature is lacking.

**Methods:**

A systematic review of the literature was performed according to PRISMA guidelines. Pubmed, Embase, Cochrane, and Medline databases were consulted using MESH keywords. Non-English written articles and abstracts were excluded. Sex, age, symptoms at presentation, diagnosis, tumor location and size, surgical approach and technique of excision, pathology, and morphology were extracted and recorded in an electronic database.

**Results:**

Sixty-seven studies for a total of 239 patients with esophageal lipoma or liposarcoma were included in the qualitative analysis. Among 176 patients with benign lipoma, the median age was 55. The main symptoms were dysphagia (64.2%), transoral polyp regurgitation (32.4%), and globus sensation (22.7%). The majority of lipomas (85.7%) were intraluminal polyps, with a stalk originating from the upper esophagus. Overall, 165 patients underwent excision of the mass through open surgery (65.5%), endoscopy (27.9%), or laparoscopy/thoracoscopy (3.6%). Only 5 (3%) of patients required esophagectomy. Of the 11 untreated patients with an intraluminal polyp, 7 died from asphyxia. Overall, liposarcoma was diagnosed in 63 patients, and 12 (19%) underwent esophagectomy.

**Conclusion:**

Esophageal lipomatous tumors are rare but potentially lethal when are intraluminal and originate from the cervical esophagus. Modern radiological imaging has improved diagnostic accuracy. Minimally invasive transoral and laparoscopic/thoracoscopic techniques represent the therapeutic approach of choice.

## Introduction

Esophageal lipomatous tumors are uncommonly diagnosed and account for less than 1% of all benign esophageal neoplasms [1–3]. They can present as an intramural submucosal mass, or as an intraluminal mass with a long and narrow pedicle covered by intact mucosa and tethered to the cervical esophagus. The pedicle is usually vascularized, can be quite mobile in the esophageal lumen, and may reach into the stomach [4–6]. It is speculated that lipomas originating from the cervical esophagus near the cricopharyngeus tend to elongate and to assume a polypoid shape due to looseness of the submucosa and the long-lasting traction effect of esophageal peristalsis [3, 5, 7]. Despite esophageal lipomas are also described and reported as fibrovascular polyp, fibrolipoma, or angiolipoma [7], these neoplasms consistently share the presence of variable amounts of mature adipocytes and fibrovascular septa [4]. Due to their indolent growth pattern [8], most esophageal lipomas are estimated to be clinically silent and are incidentally found on radiographic imaging [2, 3]. In contrast, lipomas over 4 cm in size present with dysphagia, regurgitation, and/or respiratory symptoms [9]. Recurrent and life-threatening asphyxia due to oral prolapse of a mobile intraluminal lipoma is a bizarre clinical manifestation that can lead to sudden death from asphyxia [5, 6, 10]. The diagnosis is essentially based on upper gastrointestinal endoscopy, barium esophagogram, and computed tomography [11]. Excision of esophageal lipomatous tumors can be performed extraluminally, through an esophagotomy, or endoluminally through an endoscopic approach [11–13]. Liposarcoma is extremely rare, although some neoplasms presenting as giant fibrovascular polyps have been diagnosed as liposarcomas on histopathological assessment [14].

The aim of this study was to provide a comprehensive and updated systematic literature review on the clinical-pathological features and therapeutic approach to esophageal lipomatous tumors.

## Material and methods

**Fig. 1:**
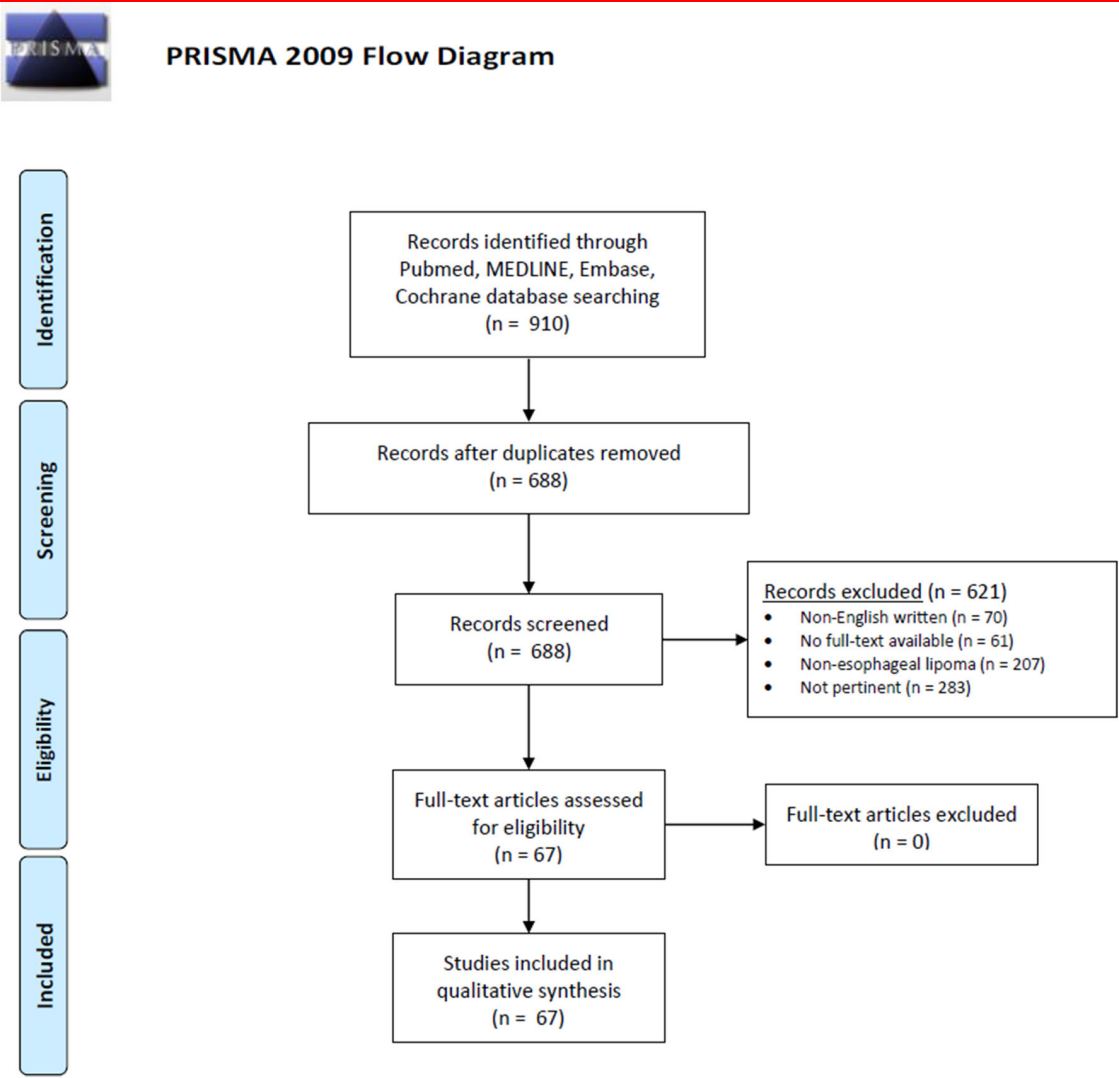
PRISMA flowchart

An extensive literature search was conducted according to the Preferred Reporting Items for Systematic Reviews and Meta-analyses (PRISMA) statement [15]. Two independent authors (DF, DB) conducted the literature search to identify all English-written reports on esophageal lipoma and liposarcoma. Pubmed, Embase, Cohcrane, and Medline databases were accessed to May 1, 2020, using Google Chrome browser (Google, California, USA). The following specific MeSH keywords were used: esophagus; lipoma; polyp; fibrolipoma; angiolipoma; liposarcoma. The references list of each selected article was consulted to broaden the search. Articles where included in the research database if they reported a case of lipoma, fibrovascular polyp, fibrolipoma, angiolipoma, or liposarcoma of the esophagus in the adult or pediatric population. Tumors proximal to the cricopharyngeus were excluded. Non-English written articles and abstracts were excluded from final analysis (Fig. 1). Three authors (DF, DB, VL) independently extracted data from the appropriate studies, including author name, year, journal of publication, number of patients, age, sex, symptoms, diagnosis, location, morphology, size, therapeutic approach, histological findings, and adverse events. The methodological quality of the studies was assessed based on the most critical factors that increase the risk of bias in this specific context [16].

Inter-rater reliability was assessed for study selection and extraction of data using Cohen’s kappa coefficient. In case of disagreements between the authors, discussion and agreement were required; if consensus was not reached, a senior author (LB) made the decision.

Continuous data are reported as median ± interquartile range (IQR) or mean ± standard deviation (SD). Categorical demographic and baseline variables are reported as proportions or frequencies. Statistical analysis included Chi-square test to compare percentages and Mann–Whitney U test to compare continuous data. A *p* value < 0.05 was considered statistically significant. Statistical analyses were performed using SPSS software 23.0 (IBM, Armonk, New York, USA).

## Results

Sixty-five case reports, [1, 2, 5, 6, 8, 10, 12, 13, 17–73] and two reviews [11, 74], published between 1955 and May 2020, were included in the systematic review. The total number of patients was 239, including 176 with lipoma and 63 with liposarcoma. All studies had a retrospective design, and a low (*n* = 59) to moderate risk (*n* = 8) for bias based on a global assessment of methodological quality [16].

### Esophageal lipoma

The median patient age was 55 (IQR 20), and the male to female ratio 2:1. The majority of tumors were intraluminal (*n* = 156, 88.6%), and were generally described as fibrovascular polyps (Table 1). The most frequent site of origin was the cervical esophagus (85.7%), followed by the distal (8%) and the mid-thoracic esophagus (6.3%). In 43 of the 65 papers, the time to first physician consultation averaged 12.2 (±10.2) months from the onset of symptoms. The most frequent symptom at presentation was dysphagia (*n* = 113), followed by polyp regurgitation (*n* = 57), and globus sensation (*n* = 40). Weight loss was reported in 32 patients, and averaged 8 kg. Respiratory symptoms were reported by 15.9% of patients and ranged from mild cough to aspiration pneumonia. Asphyxia secondary to polyp regurgitation occurred in 12 patients (6.8%). The reporting of polyp regurgitation was often impressive (“fleshy tissue extruded from the mouth”) and accompanied by spectacular pictures showing the mass in the throat or prolapsing from the mouth. Most patients admitted to re-swallow the polyp, and occasionally to capture it to convince a skeptical physician. Anemia was reported in 17 patients (9.6%) and was attributed to occult bleeding originating from the ulcerated tip of a long fibrovascular polyp (>15 cm) reaching the gastroesophageal junction and exposed to gastroesophageal reflux [12, 24, 35, 43, 58]. Physical examination was unremarkable in most patients, except those presenting with acute transoral polyp regurgitation (*n* = 3) (Table 2).

**Table 1:** Demographics and clinical characteristics of patients with esophageal lipoma

	*N*=176
Median age, years (IQR)	55 (20)
Male, *n* (%)	117 (66.5)
Location of the tumor	
Cervical esophagus, *n* (%)	150 (85.7)
Thoracic esophagus, *n* (%)	11 (6.3)
Distal esophagus, *n* (%)	14 (8)
Site of the tumor	
Intraluminal, *n* (%)	156 (88.6)
Intramural, *n* (%)	20 (11.4)
Duration of symptoms, months (± SD)	12.2 (± 10.2)
Adverse events, *n* (%)	18 (10.2)
Deaths, *n* (%)	8 (4.5)

**Table 2:** Presenting symptoms in 176 patients with esophageal lipoma

Symptom	*n* (%)
Dysphagia	113 (64.2)
Tumor regurgitation	57 (32.4)
Lump sensation	40 (22.7)
Weight loss	32 (18.2)
Respiratory symptoms	28 (15.9)
Cough	16 (9)
Asphyxia	12 (6.8)
Food regurgitation	27 (15.3)
Anemia	17 (9.7)
Chest Pain	14 (8)
Odynophagia	13 (7.4)
Heartburn	8 (4.6)

Further diagnostic evaluation included upper gastrointestinal endoscopy, barium swallow (mainly in studies published before the 1980′), or chest and neck computed tomography (CT). Endoscopy failed to detect a fibrovascular polyp in up to 33% of cases [11]. Barium swallow study usually showed a luminal narrowing at the level of the tumor. CT scan, performed in 56 patients, was consistently diagnostic and showed an hypoattenuating submucosal mass with fat density (Hounsfield units between −90 and −110) [2, 59]. Magnetic resonance was performed in 11 patients and showed a mass with hyperintense signal on T1 and T2-weighted images. Endoscopic ultrasound, performed in 13 patients, was considered especially useful to assess the presence of feeding vessels in the stalk of a fibrovascular polyp [36]. Positron emission tomography was performed in 2 patients to exclude malignancy [57, 66]. In one patient with intramural lipoma of the distal esophagus, high-resolution esophageal manometry revealed esophageal pan-pressurization, but the lower esophageal sphincter was properly relaxing upon swallowing [70].

The therapeutic approach varied depending on symptoms at presentation, location of the mass, and time of publication of the article. The most common excisional approach was through a left cervical esophagotomy (*n* = 63), followed by endoscopic resection (*n* = 46) via flexible endoscopy in 39 patients and rigid endoscopy in 7, and by open esophagotomy through a right thoracotomy (*n* = 44). Only 3% of the patients underwent a minimally invasive approach by thoracoscopy (*n* = 3, including one robotic-assisted enucleation) or laparoscopy (*n* = 3). Only 5 patients (3%) underwent esophagectomy. Of note, five (3%) of the 165 patients were operated emergently due to asphyxia secondary to polyp regurgitation, and required tracheotomy and subsequent cervical esophagotomy (*n* = 2), primary cervical esophagotomy and mass excision (*n* = 2), and endoscopic resection (*n* = 1).

Overall, 6 (3.6%) postoperative complications were reported including esophageal anastomotic leak, pneumothorax, and esophageal mucosa laceration after endoscopic resection treated with a temporary fully covered stent. One patient (0.6%) died of hemorrhage following incomplete endoscopic removal. The patients were discharged at an average of 7.8 ± 7.2 (range 0–32 days) after intervention, as reported in 36 papers. No deaths occurred among the 113 cases described after the 1980′ (*p* < 0.01) (Table 3).

**Table 3:** Therapeutic approach to esophageal lipoma in 165 patients

Therapy	*n* (%)	Morbidity	Mortality
Cervicotomy	63 (38.2)	1 (anastomotic leak)	0
Endoscopy	46 (27.9)	1 (mucosal laceration)	1 (postoperative bleeding)
Flexible	39 (23.7)		
Rigid	7 (4.2)		
Thoracotomy	44 (26.7)	1 (pneumothorax)	0
Esophagectomy	5 (3)	2 (anastomotic leak, pneumothorax)	0
Laparoscopy	3 (1.8)	0	0
Thoracoscopy	3 (1.8)	0	0
Laparotomy	1 (0.6)	0	0

**Fig. 2:**
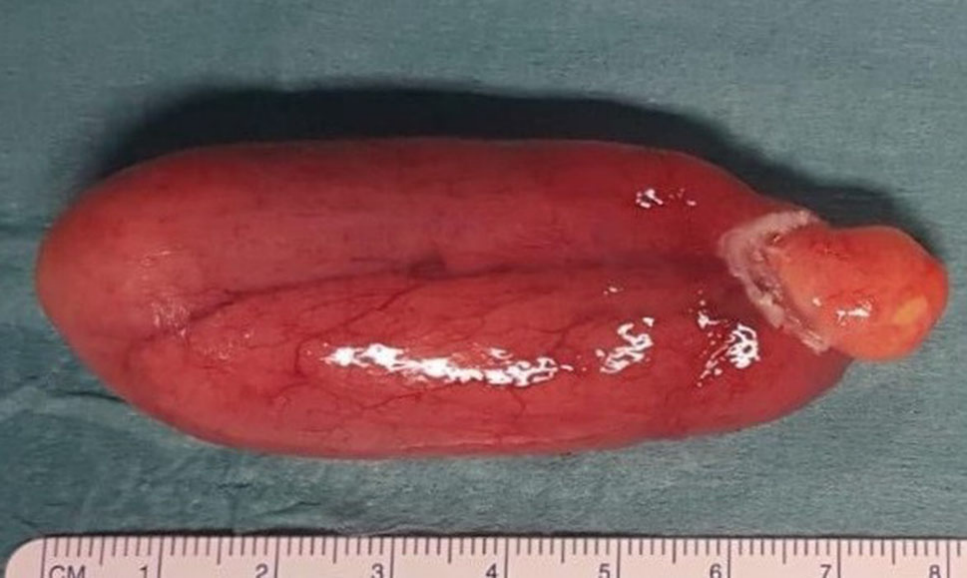
Intraluminal polyp excised with endoscopic snare technique

**Fig. 3:**
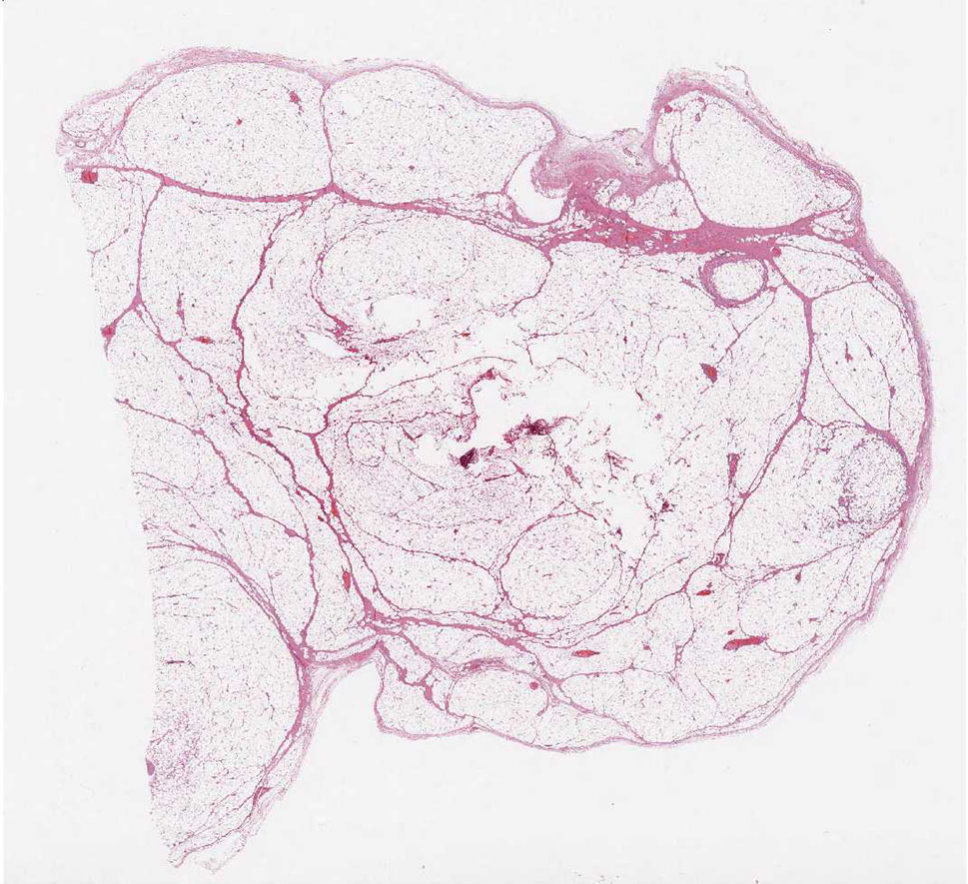
Microscopic transverse section revealing benign esophageal lipoma

All tumors were described as lipomatous benign masses with variable amount of mature adipose tissue and fibrovascular septa (Figs. 2, 3); the final pathologic diagnosis was fibrovascular polyp in 42.3%, lipoma in 33.4%, fibroma in 14.7%, fibrolipoma in 8.3% of patients, and angiolipoma in 1.3%.

**Fig. 4:**
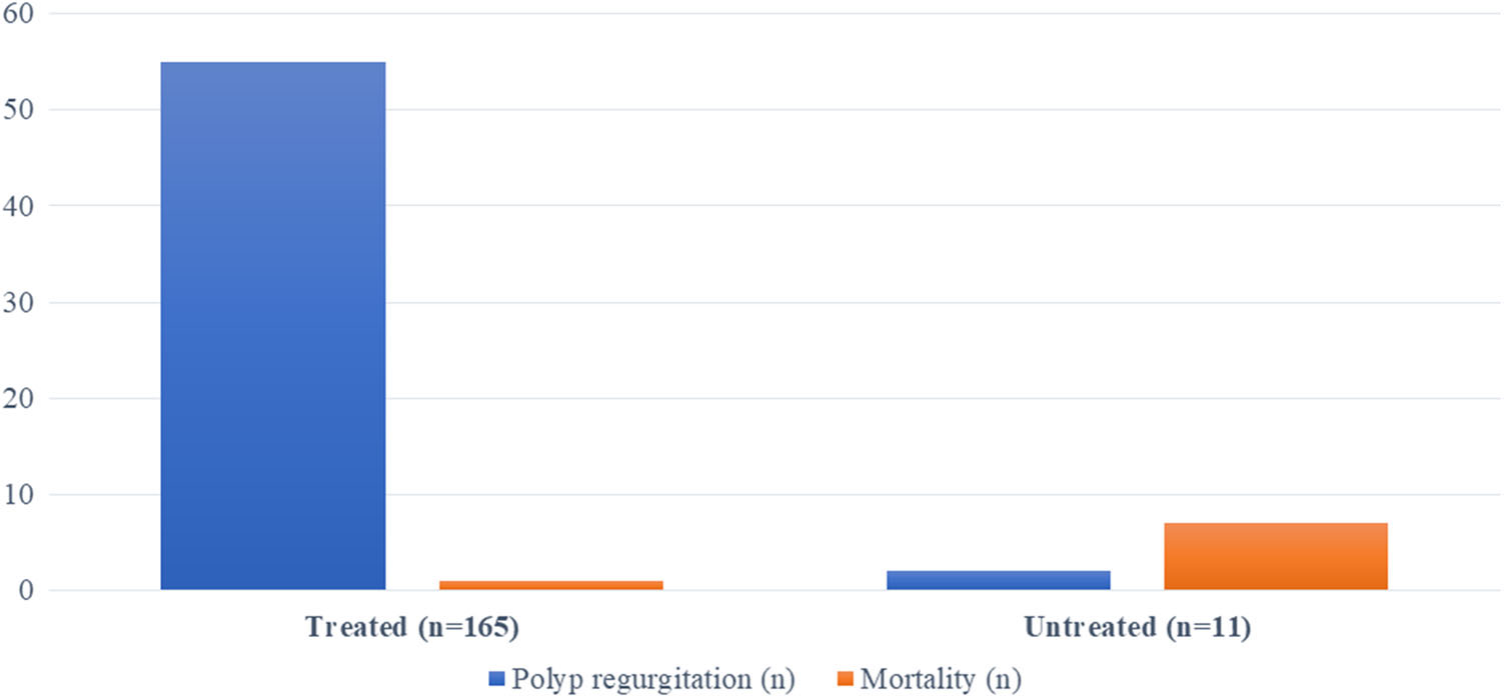
Prevalence of polyp entrapment in the throat and mortality related to asphyxia in patients treated or not treated for esophageal lipoma

Of the 11 patients who were not treated surgically or endoscopically, 7 died of acute asphyxia due to entrapment of the polyp in the throat and/or aspiration. The remaining 4 patients refused treatment; 2 of them were followed-up up to three years with CT scan which showed no increase in size of the mass (Fig. 4).

Table 4 shows a comparative analysis of intraluminal and intramural tumors. Intraluminal lipomas were significantly longer (*p* = 0.02) and arised more frequently from the cervical esophagus (*p* < 0.01), while intramural lipomas were mostly located in the distal esophagus (*p* < 0.01). Overall, adverse events and deaths occurred more commonly in patients with intraluminal polyps (*p* = 0.97).

**Table 4:** Comparison between intramural and intraluminal lipomas of the esophagus

	Intramural (*n*=20)	Intraluminal (*n*=156)	*p*
Male, *n* (%)	14 (70)	103 (66)	0.71
Age, years, mean (± SD)	54.7 (± 20.8)	55.6 (± 15.9)	0.82
Site (%)			
Cervical	9 (47.4)	141 (90.4)	
Thoracic	4 (21.1)	7 (4.5)	< 0.01
Distal	6 (31.5)	8 (5.1)	
Size (± SD)			
Length	9.46 (± 5.5)	12.5 (± 5.3)	0.02
Width	4.3 (± 2.1)	3.8 (± 2.1)	0.32
Duration of symptoms, months (± SD)	11.6 (± 8.1)	11.1 (± 9.3)	0.82
Adverse events, *n* (%)	2 (10)	16 (10.2)	0.97
Deaths, *n* (%)	0 (0)	8 (5.2)	0.30
Hospital stay, days, mean (± SD)	9.2 (± 8.3)	7.6 (± 6.7)	0.33

### Esophageal liposarcoma

Sixty-three patients with esophageal liposarcoma were found, 62 from a review [74] and one more recent case report [73]. Most liposarcomas were polypoid; in 10 patients, an endoscopic approach was used, whereas 9 patients were treated through a left cervical incision. Esophagectomy was performed in 12 patients. The main patients’ features are reported in Table 5.

**Table 5:** Demographic, clinical, and pathological characteristics of patients with esophageal liposarcoma

	*N* = 63
Median age, years (IQR)	66 (21)
Male, *n* (%)	46 (73)
Location of the tumor	
Cervical esophagus, *n* (%)	46 (73)
Thoracic esophagus, *n* (%)	8 (12.7)
Distal esophagus, *n* (%) Not reported	5 (7.9) 4 (6.4)
Site of the tumor	
Intraluminal, *n* (%)	54 (85.7)
Intramural, *n* (%)	9 (14.3)
Histology Well-differentiated, *n* (%) Myxoid, *n* (%) Pleomorphic, *n* (%) Dedifferentiated, *n* (%) Not specified	40 (63.5) 7 (11.1) 1 (1.6) 9 (14.3) 6 (9.5)
Local recurrence	6 (9.5)
Death related to disease recurrence	2 (3.2)

## Discussion

The present systematic review shows that the vast majority of esophageal lipoma is represented by fibrovascular polyps originating from the cervical esophagus. All adverse events related to the natural history of esophageal lipoma or to the consequences of therapeutic intervention occurred before the 1980′, reflecting in part limited use of radiological imaging that may have accounted for late diagnosis. In fact, five patients required emergent tracheotomy or intubation, and 7 of 11 untreated patients died of asphyxia, thus underscoring the importance of early diagnosis and endoscopic treatment [27].

After the 1980s, there has been a shift toward first-line endoscopic excision of fibrovascular polyps, and this procedure has largely replaced open cervical esophagotomy. Since the review of Caceres et al. [11], the rate of cervical lipoma approached through endoscopy has grown from 21.5 to 46% (*p* < 0.01).

Today, thanks to technological advancements, flexible endoscopy is the first-line approach even in patients with giant fibrovascular polyps. The risk of bleeding from the area of mucosal transection and from the central vascular axis of the polyp is minimal if the stalk is visible and a snare device can be easily applied. In some circumstances, a cross-over approach with rigid endoscopy could be crucial to avoid open surgery. A recent meta-analysis comparing flexible and rigid endoscopy showed that both approaches were safe and effective for retrieval of upper esophageal foreign bodies [75]. The Weerda diverticuloscope, commonly used for the treatment of Zenker diverticulum, allows to introduce a rigid 5-mm telescope along with multiple devices, such as graspers, forceps or endo-staplers [30].

**Fig. 5:**
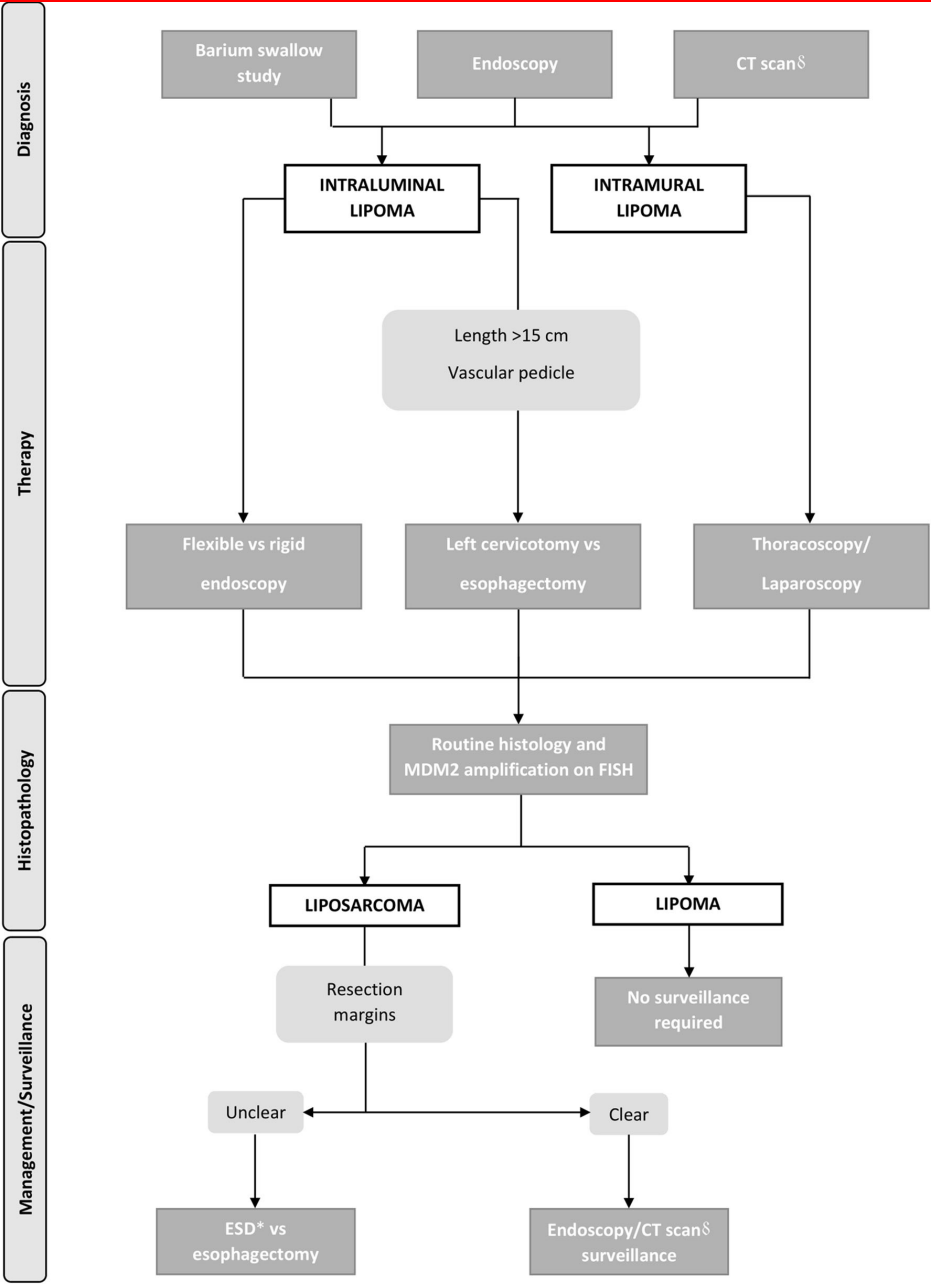
Proposed algorithm for diagnosis, treatment, histopathological assessment, and surveillance of esophageal lipomatous tumors. * ESD = Endoscopic Submucosal Dissection; δ CT scan = computed tomography scan

When the snare technique is not feasible because of the difficulty of trapping the polyp stalk or the risk of an incomplete resection, endoscopic submucosal dissection (ESD) is another option that has been shown to be safe even in patients with giant fibrovascular polyps [5, 53, 63]. Compared to the snare technique, ESD can increase the chance of a radical excision of the polyp with clear margins. In the present systematic review, there were 5 reported recurrences or malignant transformations after initial snare excision of an apparently benign lipoma. Unfortunately, the resection margin was not evaluated [11, 12]. Two studies reviewing the clinical-pathological and molecular features of esophageal lipoma and liposarcoma found MDM2 amplification by fluorescence in situ hybridization in all cases, with some of these patients presenting recurrent disease [14, 76]. Therefore, a careful histopathological assessment of the resection margin, is recommended after resection of an apparently benign lipoma, and patients should be advised about the need for endoscopic/radiologic surveillance. Given the potential malignancy of esophageal lipomatous neoplasms, we suggest an algorithm for diagnosis, treatment, and surveillance (Fig. 5).

The rarity of esophageal lipoma, the heterogeneity of reported data, the differences in histopathological assessment and classification, and the therapeutic selection bias are the main study limitations.

In conclusion, esophageal lipoma is an uncommon but potentially life-threatening condition. Clinical presentation and treatment remain heterogeneous in the literature. For polyps of the upper esophagus, early intraluminal approach by flexible or rigid endoscopy and careful histopathological assessment is recommended. Distal esophageal and intramural lesions may safely and effectively be approached by laparoscopy or thoracoscopy to reduce patient hospitalization and potential morbidity related to open surgery.
